# Pupillary light reflex in ethambutol-induced optic neuropathy

**DOI:** 10.1038/s41598-020-77160-5

**Published:** 2020-12-10

**Authors:** Yung-Ju Yoo, Jeong-Min Hwang, Hee Kyung Yang

**Affiliations:** 1 Department of Ophthalmology, Kangwon National University Hospital, Kangwon National University Graduate School of Medicine, Chuncheon, Korea; 2 Department of Ophthalmology, Seoul National University Bundang Hospital, Seoul National University College of Medicine, 82, 173 Beon-gil, Bundang-gu, Gumi-ro, Seongnam, Gyeonggi-do 13620 Korea

**Keywords:** Diseases, Neurology

## Abstract

We evaluated changes in the pupillary light reflex (PLR) of ethambutol (EMB)-induced optic neuropathy and analyzed the correlations between PLR parameters and other structural changes in EMB-induced optic neuropathy. This retrospective, observational, case–control study involved thirty-two eyes of 17 patients with EMB-induced optic neuropathy (EON group), sixty eyes of 60 patients without EMB-induced optic neuropathy (non-EON group) while taking ethambutol, and forty-five eyes of 45 normal controls. PLR was measured by digital pupillometry. The clinical characteristics, optical coherence tomography measurements and PLR parameters including pupil diameter, constriction latency, constriction ratio/velocity, and dilation velocity were noted. The differences in PLR measurements were compared among the three groups. Correlations between PLR parameters and other structural parameters in EMB-induced optic neuropathy were evaluated. The pupillary constriction ratio, constriction and dilation velocities were significantly reduced in the EON group compared to the non-EON group and controls (all *P* < 0.05). In EMB-induced optic neuropathy, average outer macular ganglion cell layer (mGCL) thickness showed a significant correlation with the pupillary constriction ratio (ß = 4.14, *P* = 0.003) and maximal constriction velocity (ß = 1.08, *P* < 0.001). This study confirmed that pupillary constriction and dilation velocities were significantly decreased in patients with EMB-induced optic neuropathy, compared to normal controls. Digital pupillometry may be a useful tool in the evaluation of EMB-induced optic neuropathy.

## Introduction

Ethambutol (EMB) is an integral first-line bacteriostatic antibiotic used in combination drug regimens for treatment of tuberculosis (TB) or *Mycobacterium avium complex* infections^1,2^. The most devastating adverse effect of EMB is toxic optic neuropathy, which occurs in 1–2.5% of patients^3^. Although EMB-induced optic neuropathy is reversible after cessation of the drug, complete recovery is not always possible, often resulting in permanent visual impairment^4^. For this reason, diagnosis of EMB-induced optic neuropathy in the early phase is important. However, diagnosis of EMB-induced optic neuropathy is especially difficult in elderly patients because other ophthalmological problems such as dry eye syndrome, cataract, or epiretinal membrane, can also cause visual disturbances in these individuals^5,6^.

One notable feature of EMB-induced optic neuropathy is that the retinal ganglion cells located in the papillomacular bundle is particularly susceptible to damage, which is related to abundant mitochondrial function of the macular ganglion cell layer (mGCL)^7–9^. The pupillary light reflex (PLR) is also a well-established method of identifying loss of ganglion cell function^10^. However, changes in the PLR of patients with EMB-induced optic neuropathy have rarely been addressed^11,12^. There has been a brief mention of pupil cycle prolongation in previous studies concerning patients on ethambutol therapy, but detailed analysis of the pupillary light reflex has not been documented^11,12^ Recently, digital pupillometry enables objective quantification of PLR parameters^13,14^. This non-invasive and simple test has been proved to be effective in diagnosing other optic neuropathies and pupillary disorders^15,16^.

In the present study, we investigated the characteristics of PLR parameters measured by digital pupillometry in patients with EMB-induced optic neuropathy. We also evaluated the correlations between PLR parameters and functional and structural measurements of the optic nerve.

## Results

### Demographics and clinical characteristics

This study included 32 eyes of 17 patients with EMB-induced optic neuropathy (EON group), 60 eyes of 60 patients without EMB-induced optic neuropathy while taking ethambutol (non-EON group), and 45 eyes of 45 healthy control subjects. The interval between onset of symptoms to initial ophthalmologic examination was 31.7 ± 16.6 days (range 7–60). The mean duration of EMB use was 189 ± 72 days (range 60–294) and mean daily dose of EMB was 18.0 ± 3.6 mg/kg (range 13.3–25.0).

The mean age of the EON group was 68.6 ± 14.5 years (range 31–86), and twelve of them (70.6%) were female. There was no significant difference in the mean age among the three groups (non-EON group, 64.6 ± 11.5 years; control group, 65.4 ± 10.5 years, p = 0.297). The mean best corrected visual acuity (BCVA) of the EON group was 1.07 logarithm of the minimum angle of resolution (logMAR) (standard error [SE], 0.09) at initial presentation, which was significantly worse than the non-EON group (mean 0.01 logMAR, SE 0.02, *P* < 0.001) and the control group (mean 0.02 logMAR, SE 0.02, *P* < 0.001).

Spectral-domain optical coherence tomography (SD-OCT) measurements were compared among groups. General circumpapillary retinal nerve fiber layer (cpRNFL) thickness was significantly thicker in the EON group than both the non-EON group and control group (*P* = 0.048 and 0.019, respectively). On the other hand, the average inner and outer mGCL thickness defined by the Early Treatment Diabetic Retinopathy Study (ETDRS) map were thinner in the EON group (38.5 and 31.1 μm) compared to the non-EON group (48.3 and 36.2 μm) and control group (50.5 and 34.7 μm) (all *P* < 0.01) (Table 1).

**Table 1:** Comparison of the pupillary light reflex and optical coherence tomography parameters among the three groups.

Parameters	EON group (32 eyes, 17 patients)		Non-EON group (60 eyes, 60 patients)		Control (45 eyes, 45 patients)		*P* value* EON vs. Non-EON	*P* value* EON vs. Control	*P* value* Non-EON vs. Control
	Mean	SE	Mean	SE	Mean	SE			
**BCVA (logMAR)**	**1.07**	**0.09**	**0.01**	**0.02**	**0.03**	**0.01**	** < 0.001**	** < 0.001**	0.520
Pupillary light reflex parameters									
Maximal pupil diameter (mm)	4.60	0.19	5.00	0.13	5.11	0.15	0.166	0.084	1.000
Minimal pupil diameter (mm)	3.63	0.28	3.56	0.25	3.67	0.25	0.951	0.746	0.782
**Pupil constriction (%)**	**28.9**	**1.28**	**34.0**	**0.76**	**35.9**	**0.79**	** < 0.001**	** < 0.001**	1.000
**Latency (s)**	**0.26**	**0.01**	**0.23**	**0.01**	**0.23**	**0.01**	**0.046**	**0.001**	0.350
**Average constriction velocity (mm/s)**	**2.79**	**0.61**	**3.55**	**0.36**	**3.55**	**0.48**	**0.014**	**0.001**	1.000
**Maximal constriction velocity (mm/s)**	**3.69**	**0.21**	**4.60**	**0.13**	**4.65**	**0.14**	** < 0.001**	** < 0.001**	1.000
**Average dilation velocity (mm/s)**	**0.69**	**0.04**	**0.89**	**0.03**	**0.86**	**0.03**	** < 0.001**	**0.001**	0.958
T75 (s)	2.08	0.18	2.25	0.13	2.37	0.15	1.000	0.313	0.856
cpRNFL thickness (μm)									
**General cpRNFL thickness**	**106.9**	**2.6**	**101.3**	**0.8**	**99.6**	**1.1**	**0.048**	**0.019**	0.442
Temporal cpRNFL thickness	81.7	4.1	76.4	1.5	75.6	1.6	0.587	0.416	0.879
mGCL thickness (μm)									
**Average Inner mGCL thickness**	**38.5**	**2.0**	**48.3**	**1.0**	**50.5**	**1.2**	** < 0.001**	** < 0.001**	0.080
**Average Outer mGCL thickness**	**31.1**	**1.0**	**36.2**	**1.0**	**34.7**	**0.7**	**0.001**	** < 0.001**	0.696

Factors with statistical significance are shown in boldface; BCVA = best corrected visual acuity; cpRNFL = circumpapillary retinal nerve fiber layer; EON = ethambutol-induced optic neuropathy; logMAR = logarithm of the minimum angle of resolution; mGCL = macular ganglion cell layer; SE = standard error; T75 = Total time taken by the pupil to recover 75% of the maximal pupil diameter.

*The generalized estimating equation was used for values accounting for intereye correlations. Both eyes were included in the analyses. Covariates were sex and presence of diabetes mellitus. *P* value by post hoc test.

### Pupillary light reflex parameters in ethambutol-induced optic neuropathy

Table 1 summarizes the measurements of the eight PLR parameters in the EON group, non-EON group, and the control group. Maximal and minimal pupil diameters revealed no significant differences among the three groups. The EON group showed a significant reduction in mean pupil constriction ratio, average constriction velocity (ACV), maximal constriction velocity (MCV), and average dilation velocity (ADV) compared to both the non-EON group and controls (all *P* < 0.05). The mean PLR latency in the EON group (0.26 s) was significantly delayed compared with the non-EON group (0.23 s) and controls (0.23 s) (*P* = 0.046, *P* < 0.001, respectively). In contrast, the non-EON group showed no significant differences of all PLR parameters in comparison to controls (all *P* > 0.3).

### Factors associated with abnormal pupillary light reflex

Factors associated with the pupillary constriction ratio, ACV, and MCV were identified using a clustered regression model. Univariate analysis revealed that thinner average inner and outer mGCL thickness, lower mean deviation on Humphrey visual field (HVF) and female gender were significantly associated with decreased pupillary constriction ratio (all P ≤ 0.05). General cpRNFL thickness and average outer mGCL thickness were significantly associated with ACV (all P ≤ 0.05). MCV revealed significant associations with female gender, general cpRNFL thickness and average outer mGCL thickness (all P ≤ 0.05). 

Multivariate analysis adjusted for age, sex and the presence of diabetes mellitus demonstrated that pupillary constriction ratio (ß = 4.14, SE = 1.40, *P* = 0.003), ACV (ß = 0.919, SE = 0.210, *P* < 0.001) and MCV (ß = 1.08, SE = 0.24, *P* < 0.001) were closely related to outer mGCL thickness.

## Discussion

In our study, we used digital pupillometry to quantify PLR abnormalities in EMB-induced optic neuropathy which has not been previously reported. Our findings have important implications for the diagnosis of EMB-induced optic neuropathy in the early phase, allowing prompt cessation of the drug to enable visual recovery. In our study, pupillary constriction ratio, average and maximal constriction velocity, and average dilation velocity were significantly decreased in EMB-induced optic neuropathy compared to asymptomatic eyes of patients treated with EMB and normal controls. While the cpRNFL thickness was significantly thicker in the EON group, the inner and outer mGCL thickness was thinner indicating macular ganglion cell loss. Structural thinning of the outer mGCL thickness was associated with decreased pupillary constriction ratio and constriction velocities.

In our study, the pupillary constriction ratio, ACV, MCV and ADV were significantly impaired in EMB-induced optic neuropathy. PLR impairment in EMB-induced optic neuropathy has been reported in two previous studies^11,12^. In a prospective study, EMB-induced optic neuropathy occurred in three patients (10%) treated with EMB and the pupil reaction cycle was prolonged only in one eye^11^. In another retrospective study, the pupil reaction cycle time increased in 72% of patients^12^. However, these studies only measured the total time of PLR, without including a detailed description on constriction latency or velocities. Digital pupillometry used in our study could quantitatively analyze different phases of the PLR which has major advantages over the previous studies in understanding the characteristics of the PLR in EMB-induced optic neuropathy.

We found that mGCL thickness was significantly thinner in patients with EON versus those of the non-EON group and controls. This significant thinning of mGCL in the early phase provided evidence to suggest that mGCL damage affects visual function in EMB-induced optic neuropathy. Regarding the results of PLR impairment, it can be inferred that EMB-induced optic neuropathy induces significant damage to macular ganglion cells including those involved in the PLR.

In our study, cpRNFL thickness was increased in EMB-induced optic neuropathy, whereas mGCL thickness was decreased in the early phase of EMB-induced optic neuropathy. This is in agreement with a previous prospective study, reporting cpRNFL swelling in patients during the early phase of EMB-induced optic neuropathy, accompanied by markedly decreased perifoveal ganglion cell-inner plexiform layer thickness^17^. Retinal ganglion cells are abundant in mitochondria^6^, and are present at high density in the perifoveal area^18^. These findings suggest that EMB-induced optic neuropathy primarily involves the retinal ganglion cells located in the macula. This leads to thinning of the retinal ganglion cell bodies in the macula and axonal swelling in the peripapillary region, which are considered as early signs of EMB-induced optic neuropathy^17^.

EMB is a metal chelator which prevents cell wall synthesis in mycobacteria by inhibiting arabinosyl transferase^19^. The structural similarity between human mitochondrial DNA and bacterial ribosome allows EMB to disturb human mitochondria as well^19^. Although the exact mechanism of EMB-induced optic neuropathy is not fully understood, it has been hypothesized that accumulation of zinc in lysosomes may inhibit lysosomal activation^20,21^ and that decreased available copper in human mitochondria may disrupt oxidative phosphorylation^22^. While both of these mechanisms provoke subsequent apoptosis of retinal ganglion cells^23^, EMB-induced lysosome dysfunction may cause nonspecific damage to all the subtypes of retinal ganglion cells (RGCs) in addition to mitochondrial dysfunction.

Regarding the structure–function relationship of retinal ganglion cells, decreased pupillary constriction ratio and constriction velocity were significantly associated with thinning of the average outer mGCL thickness. The relationship between mGCL thinning and visual function impairment is well established in glaucoma as well as in various optic neuropathies^24,25^. In our study, only the outer mGCL thickness was associated with pupillary constriction parameters, while the inner mGCL thickness revealed no such correlation. This discrepancy may be attributed to the distribution of intrinsically photosensitive RGCs (ipRGCs) in the human retina^26^. The ipRGCs are crucial for non-image forming functions including the circadian photoentrainment, sleep and PLR^26^. The cell density of ipRGCs is highest in the central area 2 to 3 mm apart from the fovea, which is the same area measured in the ETDRS outer ring of the SD-OCT^26^. This may be an explanation of why the outer mGCL thickness reflects the damage of ipRGCs better than the inner mGCL thickness. However, the function of ipRGCs can only be measured by the sustained component of the PLR using blue wavelength light^27^. In our study, PLR parameters were measured with a commercial digital pupillometry using white light which cannot directly quantify ipRGC function. To clarify the relationship between ipRGC damage and PLR impairment in EMB-induced neuropathy, further prospective studies measuring sustained pupillary responses after illumination are necessary.

The cpRNFL thickness also failed to show any correlation with pupillary light reflex parameters. Several retrospective studies have reported thinning of the temporal cpRNFL thickness in EMB-induced optic neuropathy over time^9,28^. However, cpRNFL thinning in EMB-induced optic neuropathy is not detected until three months after cessation of the drug^17^. Since cpRNFL thinning is a chronic sequela of RGC damage in EMB-induced optic neuropathy, it might be of limited value for reflecting early functional impairment.

The present study is subject to several limitations. First, this is a retrospective study performed in a single center including a relatively small number of patients. Second, the lack of normative data obtained from digital pupillometry makes it difficult to interpret PLR measurements. Thus, we included healthy control subjects for comparison. Third, PLR parameters are influenced by factors such as age, sex, and the presence of diabetes mellitus. The autonomic nervous system changes with advancing age and pupillary responses are reduced^29^. To overcome this problem, we adjusted binocularity, gender, age and diabetes in the generalized estimating equation analysis which enhanced the validity of our results. In addition, it has been recently proven that stimulus size, eccentricity, luminance, and attention affects PLR measurements^30^. In order to minimize inter-device variability, we evaluated PLR parameters of each subject using the same commercial digital pupillometry. Fourth, digital pupillometry cannot directly quantify ipRGC function. The most prominent contribution of ipRGC in the PLR is the post-illumination pupil response, which appears as a sustained constriction after the offset of high-irradiance short-wavelength light^27^. Fifth, we consistently tested the right eye first when measuring the PLR. This fixed testing sequence can produce variability. Randomizing the order of tests would have been a more legitimate approach. Finally, most of the patients were not evaluated after discontinuation of EMB. Longitudinal studies with a sufficient number of patients are mandatory to confirm long-term changes of PLR parameters in EMB-induced optic neuropathy.

In conclusion, pupillary constriction ratio and velocity were significantly decreased in the early stage of EMB-induced optic neuropathy and showed significant association with outer mGCL thickness measured by SD-OCT.

## Materials and methods

### Study subjects

We retrospectively enrolled patients who were examined during EMB medication for treating pulmonary and extra-pulmonary TB between January 2013 and December 2018 in the Neuro-ophthalmology clinic of Seoul National University Bundang Hospital. The other drugs in the treatment regimen included isoniazid, rifampicin and pyrazinamide. Patients treated for TB were divided into two groups according to their neuro-ophthalmological findings. First, EMB-induced optic neuropathy was diagnosed according to the following criteria^3,31,32^, visual symptoms which occurred only after the initiation of EMB treatment, absence of symptoms associated with optic neuritis, and meeting at least one of the main criteria or two of the minor criteria. The main criteria were (1) abnormal results in the Hardy-Rand-Rittler (HRR) color vision test, and (2) central or paracentral scotoma on the visual field test. Minor criteria were (1) visual field defects other than central or paracentral scotomas, (2) decreased visual evoked potential response or 3) pallor of the optic disc (EON group). Only patients who underwent ophthalmic examinations within 60 days after the onset of blurred vision were included. The patients included in the non-EON group were those receiving EMB medication within two months, had no subjective symptoms of visual disturbance, and were confirmed to have normal visual function by one of the two neuro-ophthalmologists (J-MH and HKY).

Subjects with a history of ocular surgery other than cataract extraction, intraocular diseases (e.g., optic disc abnormalities such as optic disc drusen, optic disc edema, or optic disc neuroretinal rim pallor not related to EMB-induced optic neuropathy; and retinal diseases such as retinal vessel occlusion or diabetic retinopathy), or neurologic diseases that could cause visual field loss were excluded. By means of thorough history taking, subjects with other causes that could provoke visual disturbances such as nutritional, hereditary, and other drug-induced optic neuropathies were excluded. To compare normal PLR values, we selected an age-matched control group from individuals with normal visual function who had performed digital pupillometry.

### Ophthalmic examinations

A thorough ophthalmic examination was performed; including BCVA, automated refraction, slit lamp biomicroscopy, and dilated fundus examination. All patients underwent measurements with the standard automated perimetry (Humphrey Field Analyzer program 30–2 full threshold; white stimulus, a size III stimulus [4 mm^2^], Carl Zeiss Meditec, Dublin, USA), or Goldmann perimetry (Haag-Streit, Bern, Switzerland) if the patient could not perform HVF due to poor visual acuity defined as 20/80 or worse. Color vision was noted using HRR performed by a well-trained technician. Retinal nerve fiber layer (RNFL) thickness was assessed using the SD-OCT (Spectralis; Heidelberg Engineering, Heidelberg, Germany). The HEYEX software version 6.0 for Spectralis OCT automatically segments boundaries of the 10 retinal layers and provides measurements of the individual retinal layer thickness^33^. The accuracy of mGCL segmentation and adequate centration on the fovea were reviewed independently by masked observers (Y-JY and HKY). For retinal thickness maps, three circular lines representing 1, 3, and 6 mm scan diameters defined by the ETDRS map were obtained^34^.

### Digital infrared pupillometry

We used an automated monocular infrared pupillometry (PLR-200 pupillometry; NeurOptics Inc., Irvine, USA) to record and analyze the PLR. Each subject was dark-adapted for three minutes before measurement. PLR was consistently measured from the right eye to the left eye. Patients were instructed to focus on a small target at least three meters away with the untested eye^35^. The light stimulus was white light with illumination wavelength of 949 nm, 180 microwatts/cm^2^ in intensity, 185 ms in duration. Frequency of pupil size recording was 32 frames per second and lasted up to five seconds.

The device recorded pupil response curves for eight PLR parameters^15,35^. The maximal pupil diameter (mm) was defined as the initial resting pupil size, and the minimal pupil diameter (mm) as the smallest pupil size at peak constriction. The pupillary constriction ratio (%) was calculated using the percentage change in pupil diameter between constriction onset and peak constriction. Latency (measured in seconds) referred to the time difference between the initiation of retinal light stimulation and the onset of pupillary constriction. Average constriction velocity (ACV, mm/s) was defined as the amplitude of pupil constriction, divided by the duration of constriction. Average dilation velocity (ADV, mm/s) is the average rate at which the pupil dilates after constriction. Maximal constriction velocity (MCV) was defined as the peak value of velocity during constriction. Total time taken for the pupil to recover 75% of its maximal pupil diameter from the peak of constriction (T75) was also measured^16^.

### Statistical analysis

Statistical analysis was performed with R free statistical software (ver. 3.4.3)^36^ and related packages, including geepack^37^, emmeans^38^, doBy^39^, and pROC^40^ package. Generalized estimating equation models accounting for sex^41^, age^42^, presence of diabetes mellitus^43^ and within-patient intereye correlations were used to examine correlations and associations between variables^37,44^. One-way analysis of variance was performed to determine if there was a statistically significant difference among the PLR of the EON, non-EON, and control groups. Subsequently, the Turkey test was used to find sources of differences. Clustered regression was performed with pupillary constriction ratio, maximal constriction velocity, and average constriction velocity as dependent variables to evaluate the relationship among several factors, including BCVA, mean deviation with HVF, cpRNFL thickness, papillomacular bundle thickness, and average inner and outer mGCL thickness. Factors with a *P*-value of 0.05 or less in univariate analysis were included as candidate variables in the multivariate analysis. For all other analyses, a *p* value < 0.05 was considered statistically significant. Except where stated otherwise, the data are presented as mean ± standard deviation values.

### Ethics approval

This study adhered to the Declaration of Helsinki and the protocol was approved by the Institutional Review Board of Seoul National University Bundang Hospital (IRB No.: SNUBH B-2007/622-115). All clinical investigations were conducted according to the principles expressed in the Declaration of Helsinki. Patient records and information were fully anonymized and de-identified prior to access by any of the authors. The ethics committee waived the requirement for informed consent.
